# On the Curative Treatment of Chronic Morbus Brightii

**Published:** 1856-04

**Authors:** 


*On the curative treatment of* Chronic *Morbus Brightii.* By Dr. HANDFIELD JONES, F. R. S., Assistant-Physician to St. Mary's Hospital. (*Medical Times and Gazette, May* 19 *and May* 26, 1855.)

"Two conclusions, of the utmost importance to us in practice, seem to me fairly derivable from the knowledge we have gained respecting Bright's disease. The first is, that the morbid condition, whether attended with hypertrophy or atrophy, is of the nature essentially of depraved unhealthy nutrition, not in any wise the result of ordinary inflammation attacking a previously healthy structure. In the atrophied kidney, I see a change just such as befalls any part that, from defect of its own vital energy, gradually decays. I have found recently just the same occurring in the pancreas. The enlarged kidney is, I am sure, frequently associated with scrofulous disease in other parts. I have seen it coexisting with tubercles and vomicæ in the lungs, and deposit of bacony matter in the spleen, of a patient who died with tertiary syphilis. The enlargement of the kidney seems to take place much in the same way as the enlargement of a gland (lymphatic), which becomes the seat of scrofulous deposit. In both cases, unhealthy plasma is organized into low celloid forms, and in much the same relation to adjacent structures. The conclusion above stated is strongly supported by the latent, insidious manner in which Bright's disease usually comes on, by the efficient causes, and by the juvantia. To the former, as essentially of debilitating character, I have only space just to allude. When hyperæmia or inflammation actually makes its appearance, other symptoms are observed (especially in the urine) than those which occur in the degenerative state alone.

"The other conclusion is that, in a great majority of cases, in which the symptoms announce degeneration of the kidney, it may reasonably be anticipated that a considerable part of the organ remains in a state which is capable of restoration more or less complete. As long as the tubes are undestroyed, we may have hopes of being able to reproduce a healthy condition of their epithelial lining; if they have perished, the attempt must be ineffectual, but at any rate can scarcely be in any case injurious. We shall, therefore, do wisely to act on the most favorable supposition, and employ all our efforts to prevent the degeneration advancing further, to repair as far as possible the damage that has been effected. The question is, how shall this be done? and to this, of course, experience alone can give a satisfactory answer.—Strongly convinced as I am that Bright's disease is not inflammatory, in any correct use of the word, but is purely a disease of depraved nutrition, I can entertain no doubt that the right method of treating it is to endeavor to improve the general vigor and power of the system, and therewith its nutrition, in every possible way. We must not be satisfied with the removal of the dropsy, and restoration to apparent safety; but we must go on in the task of corroborating the system, till the urine has recovered its healthy condition, and the blood again imparts a ruddy hue to the complexion, and the muscles are tuned to strength and vigor. I do not say that we shall always, or often, be able to do all this completely, but this is what we should perseveringly aim at; and I think we have good ground for believing that such persevering effort may make all the difference to many of our patients, between an early death and many years of tolerable comfort aud enjoyment. I have seen a patient this very day who has Morbus Brightii in a marked form, with its perilous complications of dropsy, bronchitis, and threatening cerebral symptoms. His history is that he had smallpox at an early age, and has never been well since. How different might his condition now have been, had the renal degeneration, which no doubt dated from the debility induced by the smallpox, been observed and combated many years ago."

Dr. Jones then relates five cases, three of which we give, in order to show the beneficial effects of treatment. We quite agree with him, and we will say with him, "We are watchful to detect the invasion of phthisis, we combat its progress vigorously, diligently, and often successfully; why should we not do the same with this not less formidable malady?"

Case 3.—F. P., widow, æt. 57, laundress, admitted Aug. 10th. Ill fourteen days. Abdomen and lower limbs swollen. Breath short on exertion. Pulse regular and steady. Sounds of heart natural. Clear breathing in the back. Tongue furred, with long papillæ. Bowels regular. Urine of pretty healthy color, deposits a not abundant sediment, consisting of epithelial scales (many of them fattily degenerating), mucous corpuscles, or stunted particles of renal epithelium casts either homogeneous and containing a few corpuscles, or orange colored and made up of coarsely-granular and oily matter. There was also much scattered granular matter. Much albumen was thrown down by heat and nitric acid. Reaction acid. Sp. gr. 1015.

℞ Tr. Ferri Muriat. ♏x;

Tr. Digitalis, ♏v;

Inf. Quassiæ, ℥j, ter die.

Pil. Hydr. c. Coloc., gr. v, alt. noct.

28th.—Getting on very well, swelling all gone. Urine pale, deposits a dark reddish brown sediment, consisting of casts, mostly containing much molecular oil, with some corpuscles and blood globules. There are also very numerous perfect nucleated cells scattered over the field, and much diffused granulous matter forming films. It is decidedly, but not highly, albuminous; nitric acid only forms a cloud—no flakes. Reaction highly acid.

Sept. 4th.—Pt. in mist. omiss. tr. digitalis.

11th.—Complains of being heart-sick. Bowels costive. Pt. in Mist. Mist. Rhei et Magnes., ℥j o. mane. Urine at this date was scarcely altered by nitric acid; it deposited on standing a notable sediment of reddish hue, consisting of diffused granular matter, nuclei, granular corpuscles, blood disks, and a few glomeruli. These elements were sometimes united into masses, sometimes they formed complete casts of the tubes of some length. Hydrochloric acid precipitated no uric acid.

25th.—Better a good deal, no swelling, pain in forehead, which is hot for the last three days. Pt. in Mist., Olei Morrh., ʒij ter die.

Oct. 16th.—Feels quite well, not the least swelling. Urine is wheyish, pale, sp. gr. 1014, gives no precipitate, not even increased opacity with heat and acid, but after standing some hours a very notable reddish sediment subsides, which on reboiling in great part disappears. A whitish sediment (not copious) is deposited from the urine on standing, which consists of granulous matter, nuclear corpuscles, and fragments of casts, most of them containing corpuscles and more or less oily molecular matter.

Nov. 13th.—Has remained quite well since last report. Discharged. The urine now was clear, palish, acid, sp. gr. 1018; it contained no albumen, no precipitate taking place after testing with heat and acid, and allowing the tube to stand some hours. Nitric acid precipitated a tolerable quantity of lithic acid, and when added to the concentrated urine plenty of nitrate of urea was formed. A slight sediment deposited from the urine contained a very few fattily degenerating casts, and some free corpuscles possibly of renal origin.

There can be no question, I think, that chronic degenerative mischief was going on in this patient's kidneys, when she first applied to me; and there seems good ground for the hope that this was arrested, and the functional power of the kidney, as well as its nutrition, increased, while it is certain that the general health was greatly improved and invigorated. Would these results have been attained by the administration of evacuants in any form? I think not.

Case 4.—Samuel C., æt. 48, tailor, admitted November 14th. Is tall, robust, ailing last fourteen days, suffers with pain and tightness at chest; legs swell a good deal last few days; throat has been rather sore. Has pains in loins sometimes. Pulse rather jerky, weak. Tongue a little white. Heart's sounds healthy. Never had rheumatic fever. Is not in the habit of drinking, has always had good health, but has been much confined last six months; used previously to have much exercise. Urine turbid, of smoky aspect, loaded with albumen, sp. gr. 1017, after separation of albumen by infiltration, sp. gr. the same, reaction acid; on standing it deposits a rather copious whitish sediment, consisting of corpuscles, generally small, of numerous casts mostly pale and homogeneous, containing often small corpuscles or granular matter, and blood globules. Considering that I had to do in this instance with congestion of the kidney superadded to degeneration, I had him cupped on the loins to ℥v, and gave him Pulv. Jalap. co., ʒss, o. mane, as well as Tr. Ferri Muriat., ♏xc. Acid Muriat., ♏ij in Inf. Quass. ℥j, ter die.

On the 21th he stated that his breathing was a great deal easier; the swelling of the legs was less, but was still considerable; the abdomen was less swollen; the urine was a great deal more copious. The pulse was now full and forcible. He complained of having much violent pain in the head at times. Not yet feeling satisfied that the tendency to congestion of the kidney was overcome, I thought it advisable to give him Ant. Pot. Tart. gr. ss, Potass. Acet., gr. x, in Inf. Calumb. ℥j, ter die, instead of the steel, but containing the powder.

He continued this plan till Dec. 1st, when he reported that the urine was copious; he had to rise three or four times in a night; the pulse was quick, weak; the skin cool. The effusion in the abdomen had diminished, and there was less anasacra, but he had much swelling of the legs at night and of the face in the morning. Trusting that now I might safely return to the tonics, I gave him Acidi Nitrici, ♏v ex Infus. Gent. co., ter die; and Tr. Ferri Muriat., ♏x, ter die, c. Cibis, continuing the Pulv. Jalap. co. He improved immediately; the dropsy diminished, so that the legs appeared free from swelling, except a little at night.

This treatment was continued till Jan. 16th, when some more dropsical swelling of the legs appeared, and the urine was found dark colored and highly albuminous; he complained of stiffness in the loins, and had ten days before experienced some symptoms of catarrh. The pulse was 130 in the sitting posture; tongue clean; bowels open. Not wishing, if possible, to abandon the tonics, I changed the acid and steel for Hydr. Bichloridi, gr. i, Tinct. Cinchon., ℥ij; ʒj ter die ex aquâ, with Quin. Disulph., gr. ijss, in pil. ter die.

On February 6th he had less pain of head; a slight degree of swelling of the legs had occurred at night in the last two days, but there had been none previously for three weeks. The urine continued very albuminous, but less so than it had been. I continued the pill, but combined now tr. ferri mur. with the bichloride, omitting the bark.

On the 23d there was only some trifling swelling of the legs; his aspect was pallid; the urine deposited a distinct but not abundant precipitate of albumen after testing with acid; it contained scaly epithelium, and mucous corpuscular forms in plenty, a very few casts (homogenious and corpusculated), and a few blood globules.

March 16th.—He was gaining strength; the urine was rather cloudy, deposited a slight whitish sediment, was acid, and slightly albuminous, and of white color. After treatment with nitric acid, it deposited abundance of uric cristals, sp. gr. 1015. The deposit consisted of scaly epithelium, with some enal, some doubtful blood globules, and a very few casts.

The same plan was persevered in up to April 24th, when he had been gaining strength pretty steadily; had some slight swelling of the legs, and some pain in head. The urine was very pale, and sp. gr. 1014, very slightly albuminous, and contained no casts. I now changed his quinine pill for one of Quin. Disulph., gr. i, Ferri Sulph., gr. ijss, ter die, and continued his mixture.

June 12th.—He reported himself improved, stronger a great deal and had more color. The urine was slightly turbid, contained a deposit of scaly epithelium, uric acid, oxalates in small quantity, and a very few casts, some of which were homogeneous, and others imbedded granular corpuscles. Reaction was acid. It was just clouded by $NO_5$, sp. gr. 1022.

By July 20th, the same plan being continued, there was not the least dropsical swelling, and the urine was not albuminous, but contained a good many casts.

Aug. 10th.—The mixture was discontinued; he took the iron and quinine pills alone. He continued improving, finding himself not so well if he omitted his pills.

On Sept. 7th, the urine was examined at the hospital and noted to be of good amber color and not albuminous, and the same was the case on the 28th, though he was complaining of rheumatic pain in the back.

However, careful examination of the urine at home, on October 12th, showed that after treating it with heat and acid and allowing the tube to stand quietly for several hours, there was deposited a small reddish sediment of albumen. The sp. gr. 1030; the urine was of good color, slightly cloudy; it contained a very few pale, homogeneous casts, entangling oil-molecules or corpuscles.

On November 6th, he resumed the use of quinine and sulphate of iron, which had been intermitted since September 28th; they were now given in rather larger doses, two grains of the former to three of the latter, ter die.

He gained in strength and general health, and on January 4th I discharged him, very tolerably well, though certainly with less color in his face than I could have wished. His urine, then, I noted as not albuminous.

On November 6th, the urine was clear, rather light colored, of sp. gr. 1026; deposited a very few casts, homogenous and corpuscular, and some renal corpuscles. It was very acid. It was perceptibly clouded by heat and acid; deposited much uric acid in crystals after treatment with $NO_5$. After standing some time, with addition of $NO_5$, a slight precipitate remained at the bottom.

On Dec. 1[illegible]th, which was the last close examination I made before his discharge, the urine was clear, of good color, of sp. gr. 1028; contained no casts or renal epithelium; treated with nitric acid it did not appear altered. After standing a night thus tested, there were numerous crystals of uric acid deposited, and the fluid was very slightly clouded, but this was probably from lithates, as it disappeared with heat. When the urine was concentrated and treated with nitric acid, plenty of nitrate of uria was formed.

I should apologize for the length of this case, did it not appear to me of great importance as proving the efficacy of persevering therapeutical efforts. There can be no question that the kidneys were affected seriously by degenerative disease, which I am inclined to think was of the hypertrophic kind. The influence of remedies was decided, but very gradual, and it is of especial interest to remark that it was only the tonics that procured real, steady improvement. A critical period in the history of the case was about Jan. 16th, when it was a question of abandoning tonics, and recurring to mere evacuants. Fortunately, the bichloride of mercury came in at this time with marked good effect, and enabled us to proceed with attempts of radical cure. Three weeks later I combined the chlorides of iron and mercury, continuing the quinine, and from thenceforward recovery went on uninterruptedly. The gradual but complete change which took place in the urine was very interesting; its sp. gr. increased, the albumen disappeared, the color improved, the fibrinous casts ceased to be formed, and almost every proof was afforded that the function of the kidneys was restored. I confess that had the man been a private patient, I should have insisted strongly on his taking cod-liver oil and courses of steel for many months to come, till the improved color of his face, and the absence of any return of renal symptoms had convinced me that the cure was permanent. A sea voyage would also, beyond doubt, have been productive of good, if undertaken during the summer months, and in a warm latitude. These are, however, luxuries of treatment which the hospital physician has rarely in his power.

Case 5.—As I by no means wish to make out too favorable a case, I will now mention an instance in which the same kind of treatment, though employed pretty steadily, failed to do all that I had hoped for.

Ann U., æt. 30, married, a tall, handsome-looking person, was admitted under my care, as an out-patient, January 12th. She had an easy confinement five weeks before; the legs had been swollen before the accouchement, but did not diminish in size, nor (as she stated) did the abdomen, or but little. There was distinct fluctuation to be felt in the peritoneum; the legs were a good deal swollen. Pulse large and excited. Tongue denuded and fissured. Bowels much relaxed last fortnight, twelve motions a day. Urine was pretty clear, rather pale; sp. gr. 1015; highly albuminous; contained numerous casts, homogeneous and corpusculated, one containing a glomerulus, and some renal epithelium. The sight of the right eye was very weak, but there was no visible morbid appearance. She was suckling her infant.

I gave her at first Ol. Morrh. with Tr. Ferri Mur. ter die, and a daily purge of Pulv. Jalap, co., hoping that, as the quality of the blood improved, the dropsy would decrease; but no such favorable change took place, not even after a small cupping on the loins, and with aid of a saline diuretic containing digitalis. Elaterium was tried without benefit, and matters remained *in statu quo* about a month. I then gave her that well-known excellent combination of blue pill, squill, and digitalis, which immediately caused diminution of the dropsy; and under this she went on improving for three weeks, the mouth being slightly affected. I then gave her Tr. Ferri Mur. along with the pill, and soon after omitted the blue pill altogether.

By April 6th, she had improved so far as to be able to walk round the Serpentine; the ascites had almost if not quite disappeared, but the legs were still rather swollen. I made frequent examinations of the urine.

On March 8th, I found that the sediment contained numerous uric acid crystals, a sign which I regarded as favorable from having often observed it in the urine of patients recovering from scarlatinal dropsy.

On April 29th, the urine was clear, after having let fall a slight pale precipitate consisting of numerous epithelial scales, some pus-like corpuscles, and epithelial flakes; there were scarcely any casts to be seen. Its reaction was highly acid; sp. gr. 1014; its color a light amber; it contained a notable quantity of albumen; uric acid crystals were formed on the side of the glass in which the urine stood. Although the urine was not highly acid, it was remarkable that on each successive drop of nitric acid a white cloud was formed, which again quickly disappeared, and it was not until a good deal of acid had been added that the white cloud became permanent. I have often noticed this, and believe that the explanation is to be found in the circumstance that the albumen in the urine exists combined with alkali, soda (as in the blood serum) which requires to be completely neutralized before the albumen can be precipitated. After this I gave gallic acid, gr. v, ter die, and Tr. Ferri Mur., but without any improvement in the state of the urine. The bichloride of mercury with Tr. Ferri Mur. was also tried for some time, but, though her health and strength increased, the urine remained as before. The Liquor Ferri Persesquinit., with nitric acid, was not more effectual.

By July 20th, the dropsy had almost entirely disappeared, the sight of the right eye was much improved; she could use it well, though it was not quite strong. She then went into the country, with directions to continue the use of the Tr. Ferri Mur., which she did for more than two months, and returned in the beginning of November last, much better and stronger, and with a color. There was rather some thickening of the skin of the leg than any œdema. She is now able to do her household work, and looks very well. I have not been able to induce her to continue the steel, though I have strongly recommended her doing so.

On her return from the country, in November, the urine was pale, of sp. gr. 1015, highly albuminous, gave a pale deposit consisting of epithelial scales, numerous uric acid crystals, a few homogeneous casts, and some masses of nuclear corpuscles, together with free nuclei and granular matter.

The improvement in the case, which was effected by tonics, was limited to the general system; the urine was altered very little. I am much inclined to think that the cause of the persisting albuminuria was rather a permanent change in the capillaries of the Malpighian tufts than any considerable degeneration of the renal tubes. These capillaries, in the healthy state, have the extraordinary power of filtering off mere water and salts from the blood which traverses them; in Bright's disease this power is impaired to a greater or less degree, and liquor sanguinis, more or less altered, drains off. This impairment of the filtering power of the Malpighian capillaries is not solely and constantly associated with degeneration of the renal tubes; it is found, *per se*, to constitute the essence of the affection termed chylous urine, which can be arrested by styptic and astringent remedies (such as oil of turpentine and gallic acid), and in which, after death, no degeneration of the kidney is found to exist. Now it is quite possible that though the proper renal tissue may have recovered its healthy state, and secretes a fair proportion of urea and uric acid, yet the capillary membrane of the Malpighian tufts may remain permanently damaged, and thus liquor sanguinis will be continually draining off and mingling itself with urine otherwise healthy. Somewhat of this kind I conceive the pathological condition to have been in the last-mentioned case, as I scarcely think, if there had been any considerable defect in the depuration of the blood by the kidneys, the general system would have regained so much of health and vigor. It is remarkable that although the renal disease must, beyond doubt, have been in progress during the pregnancy, yet the confinement took place without any convulsions.

I had written the above before I examined the urine on February 24th; it was pale, wheyish, deposited a good deal of scala epithelium, but few if any casts; it contained a good deal of albumen. Its sp. gr. 1015. Its general appearance led me to fear that I had been wrong in the opinion above expressed; but when I found that HCL precipitated a good deal of uric acid, and that the concentrated urine yielded with $NO_5$ a fine specimen of nitrate urea, I was confirmed in my view.—*Ranking's Abstract.*

